# 5-HT_2C_ Receptors in the BNST Modulate Contextual Fear Conditioning Without Affecting Acute Early Life Stress-Enhanced Fear Learning in Adult Rats

**DOI:** 10.3390/brainsci14121287

**Published:** 2024-12-21

**Authors:** Brianna L. Minshall, Catherine F. Wasylyshyn, Kate M. Brand, Caroline M. Bartoszek, Kennedy A. Seipel, Madeline M. Booms, Lucy C. Chappell, Amanda N. Reichert, Jacob R. Dowell, Angeles L. Buck, Henry T. Beckett, Christopher A. Lowry, Jennifer J. Quinn

**Affiliations:** 1 Department of Psychology, Center for Neuroscience and Behavior, Miami University, Oxford, OH 45056, USA; briannaminshall@gmail.com (B.L.M.); cwasylyshyn@som.umaryland.edu (C.F.W.); brandkm@miamioh.edu (K.M.B.); caroline.bartoszek@gmail.com (C.M.B.); kennedy.seipel@gmail.com (K.A.S.); mmbooms@gmail.com (M.M.B.); lucy.clarexia@gmail.com (L.C.C.); reichean@miamioh.edu (A.N.R.); jacobdowell1995@gmail.com (J.R.D.); buckal2@miamioh.edu (A.L.B.); becketht@mail.uc.edu (H.T.B.); 2 Department of Integrative Physiology, Center for Neuroscience, University of Colorado Boulder, Boulder, CO 80309, USA; christopher.lowry@colorado.edu

**Keywords:** early-life stress, post-traumatic stress disorder, serotonin, bed nucleus of the stria terminalis, stress-enhanced fear learning, SEFL

## Abstract

Background/Objectives: Rodents provide a useful translational model of fear- and anxiety-related behaviors. Previously stressed animals exhibit physiological and behavioral stress responses that parallel those observed in anxious humans. Patients diagnosed with post-traumatic stress disorder (PTSD) present with a spectrum of debilitating anxiety symptoms that result from exposure to one or more traumatic events, with individuals exposed to early adverse experiences and women having increased vulnerability for diagnoses; however, the mechanisms of this increased vulnerability remain unknown. PTSD involves a complex network of highly interconnected brain regions, including the bed nucleus of the stria terminalis (BNST). Serotonin (5-HT) release into the BNST yields an increased expression of both fear and anxiety, specifically through 5-HT_2C_ receptor signaling. The present experiment addressed whether 5-HT_2C_ receptor signaling in the BNST is necessary for the acquisition of early-life stress (ELS)-induced enhancements in adult contextual fear learning. Methods: Rats received 0 or 15 footshocks on postnatal day 17, an established model of acute ELS (aELS) that yields enhanced adult fear learning. In adulthood, rats received bilateral infusions of a vehicle, a 5-HT_2C_ receptor antagonist (RS-102221), or a 5-HT_2C_ receptor agonist (MK-212) into the BNST 15 min prior to one-footshock contextual fear conditioning in a novel context. The next day, rats were returned to the fear-conditioning context to assess their fear memory (freezing). Results: Females demonstrated aELS-induced enhancement in contextual fear learning, while males did not. BNST infusions of RS-102221 reduced contextual fear conditioning, independent of aELS condition and sex. Infusions of MK-212 had no effect. Conclusions: Taken together, these data suggest that serotonergic signaling through 5-HT_2C_ receptors in the BNST contributes to contextual fear conditioning, but not aELS-induced stress-enhanced fear learning (SEFL).

## 1. Introduction

Stress-related psychiatric disorders, such as anxiety disorders, mood disorders, and trauma- and stressor-related disorders (e.g., post-traumatic stress disorder [PTSD]), are common and represent significant socioeconomic burden. For example, PTSD, which can develop following exposure to a traumatic event [1], has a global lifetime prevalence of 5.6% among trauma-exposed individuals [2]. PTSD has a complex etiology with demographic and environmental contributions, such as age, education level, employment status, household income, marital status, and sex [2]. One important risk factor for the development of PTSD is early-life stress (ELS) [3,4,5]. ELS refers to a variety of experiences occurring (either acutely or chronically) from the prenatal period through adolescence, and includes physical and psychological abuse, neglect, parental separation, assault, and/or injury, among others. The detrimental effects of ELS often persist throughout a child’s cognitive and emotional development, and into adulthood [4,6,7,8,9].

Rodent models can help us to understand the development of stress-related psychiatric disorders, including PTSD, through the direct manipulation of stress [10,11,12]. Preclinical animal models do not aim to replicate the human condition in its entirety, but they seek to mimic symptoms or endophenotypes associated with PTSD. Although many animal models of traumatic stress exist, there are few that adequately capture the complex nature of the disorder and the individual variability observed in humans [13]. One promising avenue is provided by the stress-enhanced fear learning (SEFL) model, which provides a method for better understanding the mechanisms underlying the relationship between ELS and exaggerated stress responding in adulthood. Further, it is important to evaluate these questions with regard to sex (e.g., [14]), since females are more vulnerable to the development of PTSD, and sexual dimorphisms in brain mechanisms are likely to mediate at least some of these differences. Rats exposed to acute early-life stress (aELS) and adult fear conditioning demonstrate SEFL compared to rats that received only adult fear conditioning (i.e., no aELS) [15,16]. However, the neural mechanisms through which SEFL occurs following ELS exposure are unknown.

One mechanism underlying the stress-induced exaggeration of anxiety and fear involves reciprocal projections between the bed nucleus of the stria terminalis (BNST) and the dorsomedial or caudal part of the dorsal raphe nucleus (DR) [17]. According to this model, corticotropin-releasing hormone (CRH)-producing neurons in the BNST selectively target the dorsomedial part of the DR to activate an anxiety- and fear-related subset of serotonergic neurons. These serotonergic neurons, in turn, project to forebrain circuits to increase anxiety and fear through 5-HT_2C_ receptor signaling. Priming of the BNST with the repeated activation of CRH receptors leads to a chronic anxiety-like state [18]. Chronic CRH overexpression in the BNST increases emotional memory expression and selectively decreases type 2 CRH receptor (CRHR2) expression in the caudal dorsomedial DR [19]. Chemical lesions of the BNST prevent the behavioral consequences of uncontrollable stress [20], as does a blockade of CRHR2 selectively within the dorsomedial part of the caudal DR [21]. Specifically, the intra-DR microinjection of a CRH receptor antagonist blocks stress-induced behavioral changes when given before stress exposure, but not when given before later behavioral testing [21]. Furthermore, the intra-DR administration of CRH, in the absence of stress exposure, dose-dependently recapitulates the effects of stress exposure, inducing escape deficits and increasing fear conditioning 24 h later. This effect is specific to the injection of CRH into the caudal DR and is not evident following the microinjection of CRH into the rostral DR [21]. Further studies have revealed that these effects are mediated by CRHR2 [22].

There has been recent interest in a potential BNST contribution to the enhanced fear and anxiety observed following acute exposure to selective serotonin reuptake inhibitors [23,24,25]. Marcinkiewcz and colleagues [23] demonstrated that 5-HT from the DR *enhances* fear and anxiety through the activation of 5-HT_2C_ receptors in the BNST. It is possible, therefore, that 5-HT_2C_ receptor activation in BNST may provide a mechanism by which early adverse experience enhances fear learning in adulthood. Here, we tested the hypothesis that 5-HT_2C_ receptors within the BNST mediate acute ELS-induced SEFL in adulthood. The goal was to determine if 5-HT_2C_ receptors in the BNST are necessary during the acquisition of adult SEFL. We predicted that the infusion of the highly selective and high-affinity 5-HT_2C_ receptor antagonist 8-[5-(2,4-dimethoxy-5-(4-trifluoromethylphenylsulphonamido)phenyl-5-oxopentyl]-1,3,8-triazaspiro[4.5]decane-2,4-dione hydrochloride (RS-102221; [26]) into the anterodorsal BNST (adBNST) would eliminate the stress enhancement of fear learning observed in aELS animals and that the infusion of the high-affinity 5-HT_2C_ agonist 6-chloro-2-(1-piperazinyl)pyrazine hydrochloride (MK-212) into the adBNST would enhance fear learning in previously non-stressed animals. As previous studies suggest sex differences in the role of reciprocal connections between the BNST and dorsomedial part of the caudal DR in control of anxiety and fear responses [17], this study was powered so that potential sex differences in BNST 5-HT_2C_ receptor contributions to SEFL could be assessed.

## 2. Materials and Methods

### 2.1. Subjects

This experiment used 166 Long–Evans rats (83 males, 83 females), bred and housed at Miami University (Oxford, OH, USA; breeders supplied by Envigo [now Inotiv], Indianapolis, IN, USA). On postnatal day (PND) 2-4, litters were culled to 10 pups (5 males and 5 females, when possible) and weaned on PND 21 (day of birth is PND 0). Rats were pair-housed with same-sex littermates for the duration of the experiment, when possible. Rats were maintained on a 14:10 hr light/dark cycle (lights on at 7:00 a.m.), with all experimental procedures performed during the light portion of the cycle. Throughout each experiment, food and water were provided ad libitum in the homecage. All procedures were conducted in accordance with the *Guide for the Care and Use of Laboratory Animals* [27] and were approved by the Miami University Institutional Animal Care and Use Committee (IACUC).

### 2.2. Apparati

*Context A:* Early-life stress exposure was performed in four identical conditioning chambers (32.4 cm L × 25.4 cm D × 21.6 cm H; Med-Associates, Inc., Georgia, VT, USA) within sound-attenuating cubicles. Chambers consisted of a white, plastic back wall; aluminum sidewalls; and a clear Plexiglas^®^ ceiling and front door. The chambers were brightly lit (140 lux) and had a flat, stainless steel grid floor with an underlying pan coated in approximately 10 mL of 50% vanilla odorant (Kroger Inc., Cincinnati, OH, USA). Grid floors were wired to a shock generator and scrambler (Med-Associates, Inc., Fairfax, VT, USA).

*Context B:* Adult fear conditioning occurred in completely novel conditioning chambers in a separate room from where the early-life stress exposure occurred. Chambers were composed of black, triangular Plexiglas^®^ inserts and floors containing 18-staggered stainless steel rods (two rows, 0.5 cm vertically apart; in each row, each rod was 1.5 cm apart). The chambers were completely dark (near-infrared lighting used for video recording), and white vinegar (Kroger Inc.) served as a context odor in pans underneath the grid floor (approximately 10 mL/pan).

*Video Behavioral Recording:* Rats were continuously monitored throughout Context A and B experimentation by progressive scan video cameras with visible light filters (VID-CAM-MONO-4A: Med Associates Inc.). These cameras were connected to a computer running Video Freeze software (Version 2.7.1.107; Med. Associates Inc.) that provides automated assessment of freezing behavior [28].

### 2.3. Procedure

**Figure 1:**
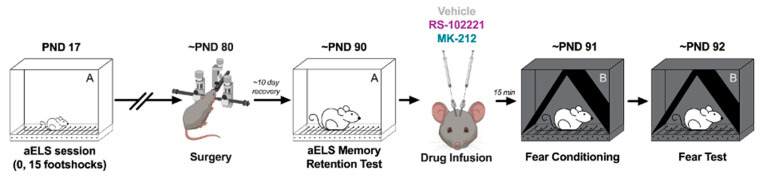
Experimental timeline. Abbreviations: acute early-life stress (aELS), postnatal day (PND). A and B indicate context designations. Image partially created using BioRender.

*aELS Session:* The early-life stress exposure occurred on PND 17 [Figure 1]. Infant rats were removed from their dams and placed with littermates into a plastic cage. The cage was transported to a laboratory and held in a room near Context A for 15 min prior to behavioral testing. Rats were placed individually into Context A for 93 min and received either 0 or 15 inescapable footshocks (1 mA, 1 s). In the 15-footshock stress exposure, the first footshock was delivered 180 s after being placed into the chamber and subsequent footshocks were delivered with a pseudorandom intershock interval of 240–480 s. Zero-footshock animals were placed into the chambers for the same amount of time in the absence of footshocks. After the stress exposure session, rats were removed from the chamber and returned to the holding room with their littermates.

*Surgery:* On PND ~80, rats were anesthetized with 5% isoflurane (Vedco, St. Joseph, MO, USA) in an oxygenated induction chamber. Rats were placed in a standard stereotaxic instrument and maintained on 2–3% isoflurane at 1 L/min. The head was leveled by equating bregma and lambda in the horizontal plane. Guide cannulae (26 gauge; P1 Technologies, Roanoke, VA, USA) were lowered into the brain bilaterally just dorsal to the BNST at stereotaxic coordinates (from Bregma: AP −0.12, ML ±2.7, DV −5.1 with a 10° angle; injection cannulae extended 2 mm beyond the guide cannula). Three to four skull screws and dental acrylic were used to close the wound. Rats received children’s liquid Tylenol^®^ (2 mg/mL) diluted in their drinking water for 72 h following surgery. Rats were allowed to recover for 7–10 days before behavioral testing began.

*aELS Memory Retention Test:* On PND ~90, rats were transported in their homecage to the laboratory and held in the same room as on PND 17 for 15 min prior to behavioral testing. Rats were individually transported to Context A as before and placed in the boxes for 8 min in the absence of footshocks to assess fear memory for Context A.

*Adult Fear Conditioning and Testing:* On PND ~91, rats were transported in their homecages to a dark holding room in the laboratory and left undisturbed for 15 min prior to behavioral testing. All cages were covered with light-eliminating shields during transport. Prior to fear conditioning, animals were restrained by an experimenter while receiving a 0.25 µL bilateral infusion of either a vehicle (40% DMSO, 60% aCSF), RS-102221 (5-HT_2C_ receptor antagonist; 2 mg/mL; [29]; Catalog #: 1050, Tocris Bioscience, Minneapolis, MN, USA), or MK-212 (5-HT_2C_ receptor agonist; 0.1 mg/mL; [30,31]; Catalog #: 0941, Tocris Bioscience) at a rate of 0.1 µL/min for a total of 2.5 min. Following infusion, all animals underwent a 2 min diffusion period before injector needles were withdrawn. All animals received 1-footshock fear conditioning (1 mA; 1 s) in a novel context (Context B) 15 min following the end of the infusion. Rats were transported to Context B in individual, blackened plastic containers (18 cm L × 32 cm D × 9 cm H). For context testing, rats were transported to Context B as they were before the day before and placed into the fear-conditioning chambers for a 5 min footshock-free session to assess fear memory for Context B.

*Euthanasia:* Following behavioral testing, rats were anesthetized with 0.2 mL Euthasol i.p. (Virbac Animal Health, Inc., Westlake, TX, USA; 390 mg pentobarbital sodium + 50 mg phenytoin sodium per mL). Rats received an infusion of a concentrated thionin stain through each cannula, using identical infusion parameters as those used the day before. Rats were perfused intracardially with 0.9% saline followed by 10% formalin. Brains were removed and placed into a 30% sucrose/10% formalin solution.

*Histology:* At least two days following perfusion, brains were frozen and sliced in 50 μm coronal sections. Every slice through the BNST was collected and mounted onto microscope slides. The brain slices were stained with 0.5% cresyl violet (Sigma-Aldrich, Inc., St. Louis, MO, USA) and then coverslipped. Infusion locations were verified using a light microscope.

*Data Analysis:* If two animals of the same sex from the same litter were in the same condition, data were averaged to obtain a single data point. A repeated measures ANOVA (rmANOVA) was performed using sex as a between-subjects factor and footshock number as a within-subjects factor to determine if there were differences in the activity burst (measured during the 1 s footshock) or postshock freezing (measured 30 s after shock) during the aELS session on PND 17. A factorial ANOVA was conducted using aELS and sex as between-subjects factors to analyze freezing during the aELS memory retention test. Separate factorial ANOVAs were conducted using aELS, sex, and acquisition drug infusion as between-subjects factors to analyze the baseline freezing, activity burst during the fear-conditioning footshock, and postshock freezing during the 30 s period following footshocks during the fear-conditioning session in Context B. An rmANOVA was performed using aELS, sex, and acquisition drug infusion as between-subjects factors to analyze freezing across the 5 min test session in Context B. Each statistical analysis was conducted using SPSS version 28.0 and at a two-tailed α = 0.05. Pairwise comparisons following a significant omnibus *F*-test or for a priori predictions were performed using Fisher’s LSD with α = 0.05. Figures were made using Graphpad Prism (version 10.3.1) and BioRender.

## 3. Results

### 3.1. Histological Verification

**Figure 2:**
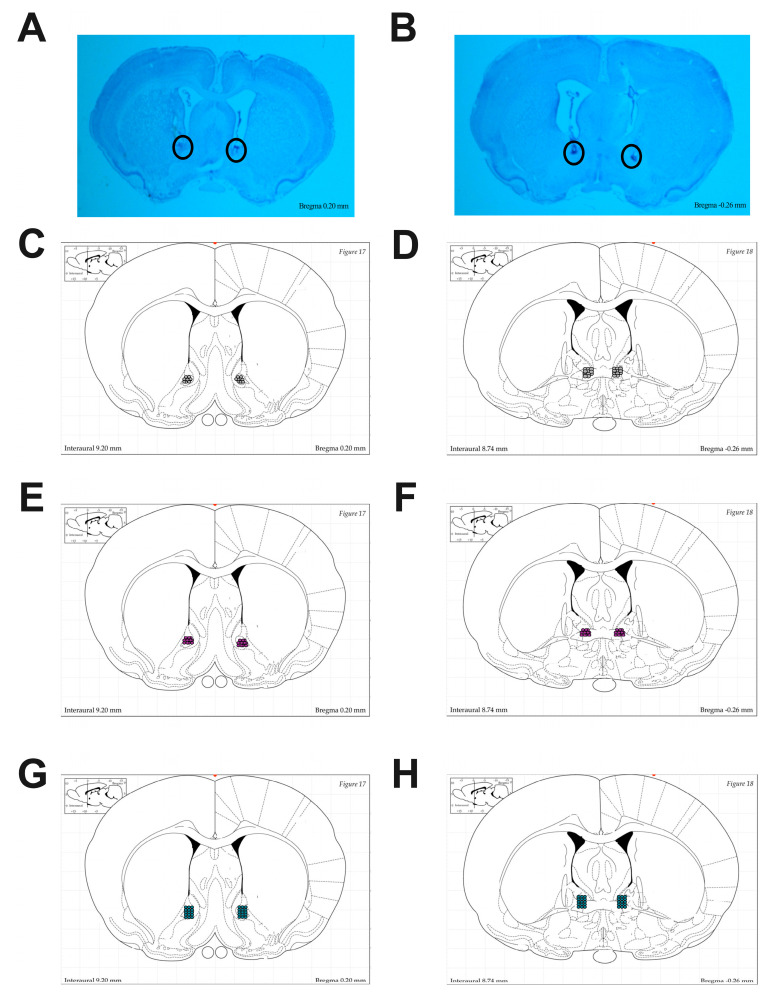
(**A**,**B**) Images depicting representative bilateral infusion placements in BNST at AP 0.2 mm and –0.26 mm relative to bregma. Atlas images depicting each infusion placement for vehicle animals (**C**,**D**), RS-102221 animals (**E**,**F**), and MK-212 animals (**G**,**H**). (**C**–**H**) Atlas images taken from Paxinos and Watson [32].

Rats with cannula placements that did not successfully target the BNST were excluded. Of the original 166 animals, 137 remained in the study following histological verification (*n* = 27 misses) [Figure 2] and equipment failure (*n* = 2). The final number in each condition was as follows: no aELS/vehicle/male = 12; no aELS/vehicle/female = 14; no aELS/RS-102221/male = 13; no aELS/RS-102221/female = 13; no aELS/MK-212/male = 13; no aELS/MK-212/female = 11; aELS/vehicle/male = 10; aELS/vehicle/female = 9; aELS/RS-102221/male = 10; aELS/RS-102221/female = 11; aELS/MK-212/male = 10; and aELS/MK-212/female = 11.

### 3.2. aELS Session

**Figure A1:**
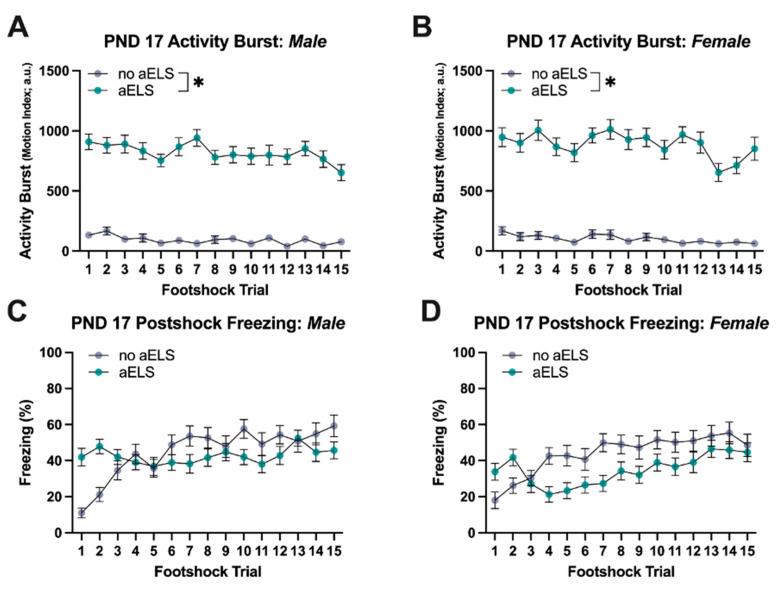
(**A**,**B**) Activity bursts (SEM) measured during each footshock of the aELS stress exposure session on PND 17. * indicates a difference between non-aELS and aELS groups. (**C**,**D**) Postshock freezing (SEM) measured during the 30 s period following each footshock of the aELS exposure session on PND 17. Abbreviations: acute early-life stress (aELS), postnatal day (PND).

During the aELS session on PND 17, there was a significant main effect of the footshock trial [F_(14, 1862)_ = 5.57, *p* < 0.001] and a significant main effect of aELS [F_(1, 133)_ = 528.15, *p* < 0.001] on the activity burst, where male and female animals that received footshocks had a higher activity burst compared to non-aELS animals [Appendix A Figure A1A,B]. Further, there was a significant footshock trial x aELS interaction [F_(14,1862)_ = 2.02, *p* < 0.05] and a significant footshock trial x sex interaction [F_(14, 1862)_ = 1.82, *p* < 0.05] on the activity burst. No other main effects nor interactions were reliable. There was a significant main effect of the footshock trial [F_(14, 1862)_ = 11.28, *p* < 0.001] on postshock freezing during the 30 s period following each footshock on PND 17 [Appendix A Figure A1C,D]. Further, there was a significant footshock trial x aELS interaction [F_(14,1862)_ = 8.25, *p* < 0.001] on postshock freezing. No other main effects nor interactions were reliable.

### 3.3. aELS Memory Retention Test

**Figure 3:**
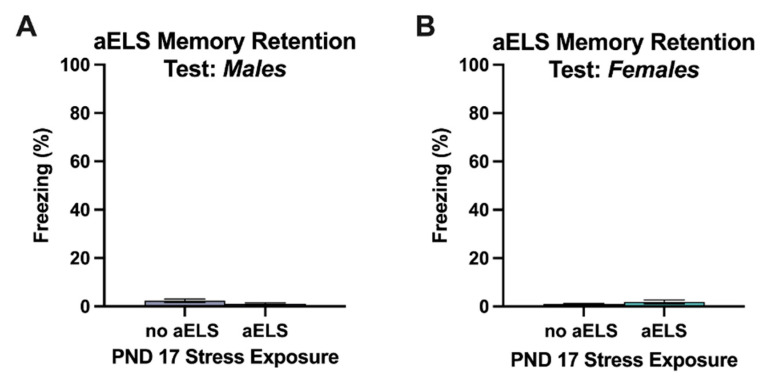
Percentages of time spent freezing (± SEM) during the aELS memory retention test during adulthood for (**A**) males and (**B**) females. Abbreviations: acute early-life stress (aELS), postnatal day (PND).

Freezing during the adult memory retention test in the aELS context (Context A) was very low (mean percent time spent freezing < 4%) in all groups. There were no significant main effects of aELS nor sex on freezing during the aELS memory retention test in adulthood. Further, there was no significant aELS x sex interaction [Figure 3].

### 3.4. Adult Fear Conditioning

**Figure 4:**
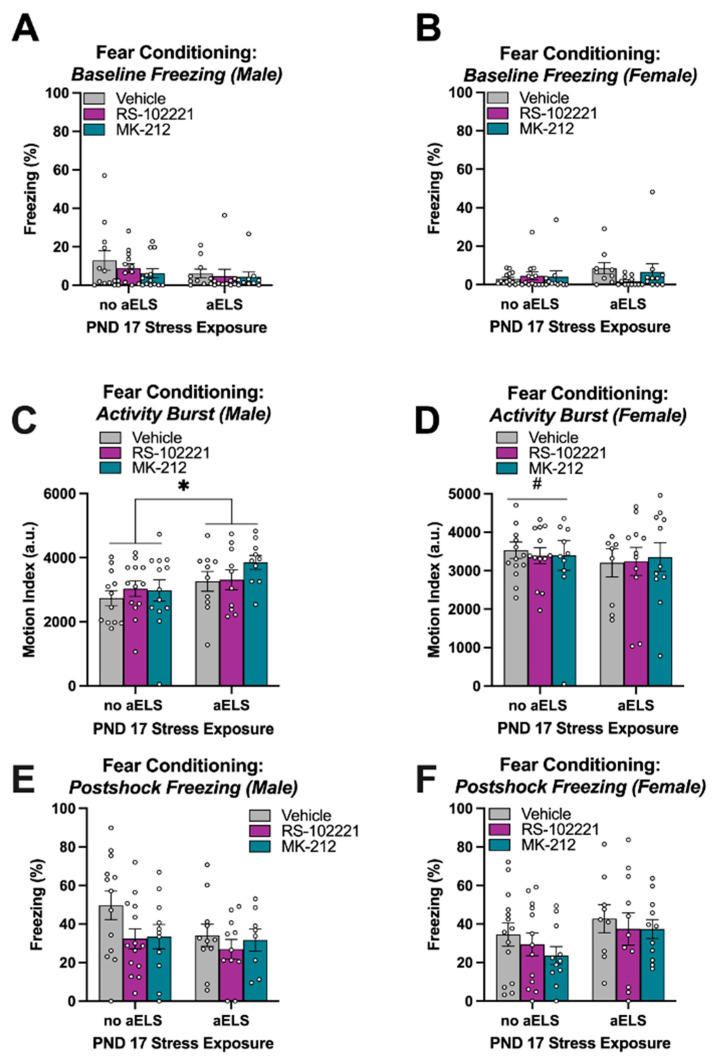
Percentages of time spent freezing (±SEM) for (**A**) males and (**B**) females during the 3 min baseline period of the adult fear-conditioning session in Context B. Activity bursts (±SEM) measured during the 1 s footshock for (**C**) males and (**D**) females. * indicates that non-aELS males had a lower activity burst compared to aELS males. ^#^ indicates that non-aELS females had a higher activity burst compared to non-aELS males. Postshock freezing (±SEM) measured during the 30 s period following footshocks of the adult fear-conditioning session for (**E**) males and (**F**) females. Data represent means ± SEMs. Individual data points are plotted on top of vertical bar graphs. Abbreviations: acute early-life stress (aELS), postnatal day (PND).

There were no significant main effects of aELS, sex, nor acquisition drug infusion on baseline freezing during the first three minutes of the adult fear conditioning in Context B. Further, there were no significant interactions on baseline freezing [Figure 4A,B]. There was a significant aELS × sex interaction on the activity burst during the 1 s footshock of the adult fear-conditioning session [F_(1, 125)_ = 4.45, *p* < 0.05]. Pairwise comparisons (Fisher’s LSD; *p* < 0.05) showed that non-aELS females had a higher activity burst compared to non-aELS males, and aELS males had a higher activity burst compared to non-aELS males; however, no difference was observed between non-aELS females and aELS females [Figure 4C,D]. No other main effects nor interactions on the activity burst were significant. There was a significant aELS x sex interaction on postshock freezing during the 30 s period following the footshock [F_(1, 125)_ = 6.20, *p* < 0.05]. However, pairwise comparisons using Fisher’s LSD (*p* < 0.05) revealed no significant difference between groups [Figure 4E,F].

### 3.5. Adult Fear Test

**Figure 5:**
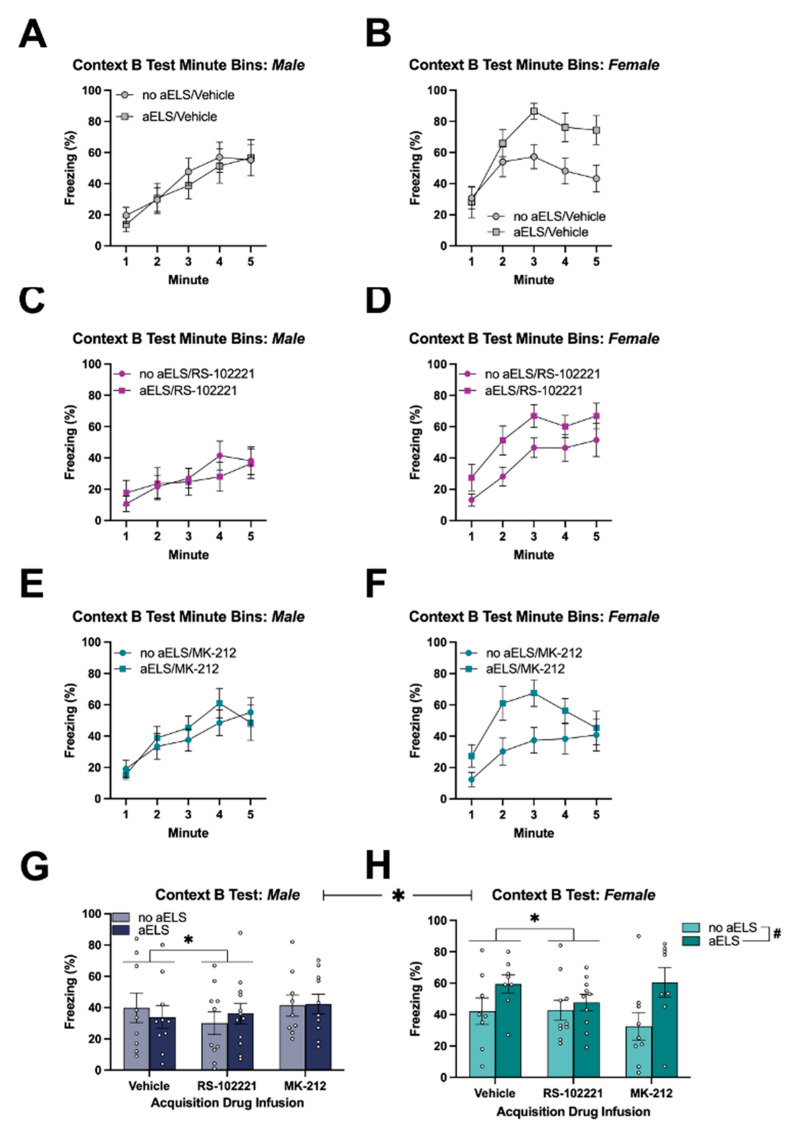
Percentages of time spent freezing (±SEM) during the 5 min test session in Context B for (**A**) males and (**B**) females that received a vehicle infusion; (**C**) males and (**D**) females that received RS-102221 infusion; and (**E**) males and (**F**) females that received an MK-212 infusion. Average time spent freezing (±SEM) during the 5 min test session in Context B for (**G**) males and (**H**) females. * indicates that animals that received an infusion of RS-102221 had lower freezing compared to animals that received a vehicle infusion. ^#^ indicates that females that received aELS had higher freezing compared to non-aELS animals. Data represent means ± SEMs. Individual data points (**G**,**H**) are plotted on top of vertical bar graphs. Abbreviations: acute early-life stress (aELS).

Across the 5 min test session in Context B, there was a significant main effect of time [F_(4, 500)_ = 66.54, *p* < 0.001] with freezing increasing across time. Additionally, there was a significant time x sex interaction [F_(4, 500)_ = 6.48, *p* < 0.001]. There were no other significant interactions with time. There was a significant main effect of aELS [F_(1, 125)_ = 5.38, *p* < 0.05] with animals that received aELS having higher freezing compared to non-aELS animals (i.e., SEFL). There was a significant main effect of sex [F_(1, 125)_ = 9.87, *p* < 0.01] with females having higher freezing compared to males. Further, there was a significant aELS × sex interaction [F_(1, 125)_ = 6.30, *p* < 0.05]. Pairwise comparisons (Fisher’s LSD; *p* < 0.05) showed that females that received aELS had higher freezing than all other groups, which did not differ from one another. Additionally, there was a significant main effect of acquisition drug infusion [F_(1, 125)_ = 3.12, *p* < 0.05]. Pairwise comparisons (Fisher’s LSD; *p* < 0.05) showed that animals that received the bilateral infusion of the 5-HT_2C_ receptor antagonist, RS-102221, had lower freezing compared to animals that received a vehicle infusion. There were no differences observed in animals that received the bilateral infusion of the 5-HT_2C_ receptor agonist, MK-212. No other between-subjects interactions were significant [Figure 5A–H].

## 4. Discussion

The present experiment aimed to address a role for 5-HT_2C_ receptor signaling within the BNST in fear acquisition in adult rats following aELS exposure in a model of SEFL. We demonstrated that early-life exposure to 15 footshocks enhanced fear learning in adulthood (i.e., SEFL), as shown previously, although this effect was driven by the females. Males did not show an aELS-induced enhancement in adult fear conditioning. Further, 5-HT_2C_ receptor antagonism (via infusion of RS-102221) in the BNST reduced adult fear conditioning, independent of sex and aELS exposure. The infusion of the 5-HT_2C_ receptor agonist, MK-212, had no effect. This demonstrates that BNST serotonergic signaling through the 5-HT_2C_ receptor contributes to adult fear learning, but not the aELS-induced stress enhancement of that fear learning.

Overall, we observed an enhancement in adult fear conditioning following exposure to aELS; however, this effect was entirely driven by the females. We previously demonstrated aELS-induced SEFL in both males and females following 15 footshocks on PND17 [14]. However, we have seen that in experiments involving surgery (gonadectomy, intracranial), male rats often do not exhibit adult SEFL following 15 infant footshocks [14]. It is possible that 15 infant footshocks is near-threshold for inducing adult SEFL in males; yet, the threshold is much lower (e.g., 4 footshocks) in females [14]. This would explain the inconsistent findings in males, though further investigation is necessary to ascertain this possibility. Of course, such threshold differences between females and males could be driven by organizational effects of gonadal steroid hormones or differences in gene expression based upon a sex chromosome complement [33]. Ongoing studies in our laboratory aim to address these questions using a “four-core genotypes” transgenic mouse model (e.g., [34,35]).

It is important to recognize that adult SEFL following infant footshock exposure is demonstrated despite a complete lack of associative fear memory for the aELS session once the animals reach adulthood [13,14,36]. Such forgetting from infancy to adulthood in preweaning animals has been used as a model of infantile amnesia [37,38,39].

The reduction in adult fear conditioning following RS-102221 infusion bilaterally into the BNST was observed during the test session 24 h following fear conditioning. There was no effect of the antagonist during the adult fear-conditioning session, suggesting that 5-HT_2C_ receptor antagonism does not impact footshock sensitivity or short-term memory of fear (also see [40,41]). Rather, 5-HT_2C_ receptor signaling in the BNST likely contributes to the consolidation of contextual fear memories, consistent with other studies demonstrating the serotonergic modulation of fear learning [42]. Interestingly, repeated exposure to prior immobilization stress in adult mice enhanced the consolidation of auditory fear conditioning (i.e., SEFL); 5-HT_2C_ receptor antagonism in the basolateral amygdala (BLA) disrupted the stress enhancement of fear learning but had no effect on fear learning in non-previously stressed mice [41]. Taken together with the present findings, this suggests a double dissociation in the role for 5-HT_2C_ receptor signaling within the amygdala and extended amygdala. However, it remains possible that this distinction represents a difference between auditory and contextual fear memory consolidation. Consistent with this possibility, it is worth noting that post-training electrolytic lesions of the BNST have been shown to disrupt the expression of contextual, but not auditory, fear memories [43], while pre-training ibotenic acid lesions have no effect on auditory fear conditioning (contextual fear memory was not assessed) [44]. Of course, such pre-training manipulations allow for potential compensatory mechanisms to support fear conditioning in the absence of the BNST. Another possible explanation for the double dissociation in the role for 5-HT_2C_ receptor signaling within the amygdala and extended amygdala could be differential mechanisms mediating the impacts of adult versus infant stress exposure. Further studies are needed to fully elaborate our understanding of serotonergic contributions to fear memory consolidation and its stress enhancement.

5-HT_2C_ receptors have been previously implicated in fear conditioning. Both systemic injection [45] and direct BNST infusion [25] of a 5-HT_2C_ receptor antagonist have been shown to block SSRI-induced increases in auditory fear memory expression in male rats. Specifically, [25] showed that the systemic injection of the SSRI citalopram enhanced fear conditioning, and the infusion of RS-102221 into the anterodorsal BNST eliminated this enhancement. This suggests that SSRIs target a subset of neurons that express the 5-HT_2C_ receptor within the BNST to enhance fear learning. Importantly, these data, along with the present findings, suggest that enhancements in fear learning that result from SSRI administration versus prior stress exposure may be mediated by separate mechanisms, since our data demonstrate that BNST infusions of RS-102221 do not disrupt aELS-enhanced fear learning. Although, once again, this difference could also result from a distinction between contextual and auditory fear conditioning, or enhancements in fear learning induced by manipulations in adulthood versus infancy.

Although more research is needed to understand the neural circuits through which 5-HT_2C_ receptors in the BNST enhance fear learning, studies by Marcinkiewcz and colleagues [23] have shown that serotonergic input to the BNST activates 5-HT_2C_ receptors that are expressed on local, non-projecting, CRH neurons. The activation of these CRH neurons by 5-HT_2C_ receptor signaling in turn enhances anxiety and fear responses by (1) activating inhibitory, putatively GABAergic projection neurons that project to the ventral tegmental area (VTA) and lateral hypothalamus (LH); and (2) the inhibition of intra-BNST, putatively anxiolytic and stress-buffering, CRH neurons that also project to the VTA/LH. Thus, overall, the activation of 5-HT_2C_ receptors in the BNST interferes with ongoing anxiolytic and stress-buffering mechanisms.

Taken together, these results speak to the heightened female vulnerability for PTSD-like symptomatology along with an important modulatory role for BNST serotonergic signaling in fear memory consolidation. These data are consistent with the model suggesting that CRH-producing neurons in the BNST selectively target the dorsomedial part of the DR to activate an anxiety- and fear-related subset of serotonergic neurons. These serotonergic neurons then project to forebrain circuits, including the BNST, to increase anxiety and fear through 5-HT_2C_ receptor signaling [17]. However, this circuit appears critical in mediating contextual fear conditioning, irrespective of prior stress history. Future studies are needed to determine the precise mechanisms through which stress-induced enhancements in contextual versus auditory fear conditioning occur and the extent to which the impacts of adult versus infant stress exposure are mediated through similar mechanisms. Overall, these studies are needed to better understand the role of the BNST in maladaptive fear in order to develop more effective therapeutic approaches for individuals diagnosed with PTSD and other fear/anxiety-related disorders.

## 5. Conclusions

Taken together, these results speak to the heightened female vulnerability for PTSD-like symptomatology along with an important modulatory role for BNST serotonergic signaling in fear memory consolidation. These data are consistent with the model suggesting that CRH-producing neurons in the BNST selectively target the dorsomedial part of the DR to activate an anxiety- and fear-related subset of serotonergic neurons. These serotonergic neurons then project to forebrain circuits, including the BNST, to increase anxiety and fear through 5-HT_2C_ receptor signaling [17]. However, this circuit appears to be critical in mediating contextual fear conditioning, irrespective of prior stress history. Future studies are needed to determine the precise mechanisms through which stress-induced enhancements in contextual versus auditory fear conditioning occur and the extent to which the impacts of adult versus infant stress exposure are mediated through similar mechanisms. Overall, these studies are needed to better understand the role of the BNST in maladaptive fear in order to develop more effective therapeutic approaches for individuals diagnosed with PTSD and other fear/anxiety-related disorders.

## Data Availability

The original data presented in the experiment will be openly available in Scholarly Commons @ MiamiOH (http://hdl.handle.net/2374.MIA/7005 (accessed on 18 November 2024)).
